# 地塞米松对顺铂诱导中国人肺腺癌原代细胞凋亡的影响

**DOI:** 10.3779/j.issn.1009-3419.2010.01.05

**Published:** 2010-01-20

**Authors:** 满香 尹, 成文 张, 勇 杨, 雪平 林, 达龙 吴

**Affiliations:** 1 314000 嘉兴，武警浙江省总队医院病理科 Department of Pathology, Zhejiang Corps Hospital, Chinese People's Armed Police Force, Jiaxing 314000, China; 2 314001 嘉兴，嘉兴学院医学院 Medical School of Jiaxing University, Jiaxing 314001, China; 3 314000 嘉兴，武警浙江省总队医院检验科 Clinical Laboratory, Zhejiang Corps Hospital, Chinese People's Armed Police Force, Jiaxing 314000, China

**Keywords:** 肺肿瘤, 顺铂, 地塞米松, Lung neoplasms, Cisplatin, Dexamethasone

## Abstract

**背景与目的:**

糖皮质激素如地塞米松能诱导淋巴细胞的凋亡并能防治因化疗引发的恶心及呕吐。然而，最近的研究资料显示糖皮质激素能引发欧洲人肺癌治疗耐药。中国人肺腺癌化疗时地塞米松对顺铂抑制肺腺癌原代细胞增殖效应的影响，目前尚不清楚。

**方法:**

分离10例经病理学确诊为中国人源肺腺癌的原代细胞，经不同浓度的顺铂+/-地塞米松处理，采用四氮唑蓝比色法（MTT）检测肺腺癌原代细胞的增殖状况，并应用流式细胞仪检测顺铂联合地塞米松处理后的人肺腺癌细胞株A549的细胞凋亡率。

**结果:**

在10例肺腺癌原代细胞中，地塞米松均可降低顺铂抑制肺腺癌原代细胞增殖的效应。同样，人肺腺癌细胞株A549，经顺铂联合地塞米松培养2周和3周后，地塞米松能降低癌细胞因顺铂诱导的细胞死亡率和凋亡率，提示地塞米松对顺铂诱导的细胞凋亡有抑制效应。

**结论:**

地塞米松可以降低顺铂诱导肺腺癌细胞凋亡的作用，建议在肺腺癌化疗时谨慎使用地塞米松。

## Abstract (en)

**Background and objective:**

Glucocorticoids, such as dexamethasone (DEX), have been widely used in conjunction with cancer therapy due to inducing apoptosis in lymphoid cells and preventing nausea and vomiting. However, recent research indicates induction of therapy-resistance by glucocorticoids in lung cancer in European, although it is unclear whether DEX mediates the resistance of cisplatin-induced apoptosis in primary cells from resected Chinese human lung adenocarcinoma specimens.

**Methods:**

Ten primary cells from resected Chinese human lung adenocarcinoma specimens were treated with cisplatin in the presence or absence of DEX. The number of viable and dead cells was evaluated by trypan blue exclusion; the cell proliferation was measured by the MTT-assay; the cell apoptotic rate was analyzed by flow cytometry.

**Results:**

In this study, it was found that DEX reduced the effect of cisplatin-induced proliferation inhibition in all of the primary cells from 10 examined tumor samples. On the other hand, the cell death and apoptosis induced by cisplatin in human lung adenocarcinoma cells line A549 could also be inhibited in the presence of DEX during 2 and 3 weeks' co-incubation.

**Conclusion:**

DEX may reduce the effect of chemotherapy with cisplatin, and it urges carefully reconsideration of the application of DEX in treatment of patients with lung adenocarcinoma in China.

过去50年以来，肺癌逐渐成为主要的癌症死亡原因。虽然对肺癌治疗方法有了很大的改进，但肺癌的远期生存率依然很低。该病预后差，约80%的患者在确诊后一年内死亡^[1, 2]^。据美国癌症协会估计，90%以上肿瘤患者的死亡受不同程度的耐药影响。糖皮质激素如地塞米松（dexamethasone, DEX）被引入肿瘤治疗的原因包括：①对淋巴细胞有促凋亡的作用；②在治疗因肿瘤引发的水肿、炎症、疼痛及电解质失衡等方面起着积极的作用；③能减缓因肿瘤药物细胞毒性作用所引发的恶心和呕吐^[3-5]^。Herr等^[6]^报道了DEX能降低因抗癌化疗药物顺铂（cisplatin, CIS）诱导肺腺癌细胞株P693细胞内凋亡基因量的表达，并提高抗凋亡蛋白基因量的表达，使得CIS联合DEX的P693细胞生长速度比单独由CIS处理的P693细胞快得多。随后，其研究组对欧洲的肺癌原代细胞接受CIS联合DEX处理后发现，DEX能降低CIS诱导细胞凋亡的效果^[7]^。这一结果与早些时候观察到的DEX对淋巴细胞有促进凋亡作用相反^[8]^。而在我国肺腺癌化疗时，常规化疗方案中都含有一种铂类药物。因此，目前有必要评估DEX对肺腺癌细胞接受铂类药物如CIS的影响。本实验收集了自2008年3月-2008年9月间入住武警浙江省总队医院、并经病理学确诊为肺腺癌的10例中国人患者的肿瘤切除标本，并分离出其原代肺腺癌细胞，参照化疗药物CIS的临床浓度，经联合不同浓度DEX处理的细胞进行体外培养观察，以模拟肺腺癌细胞接受CIS化疗联合DEX治疗的效果。因原代细胞不适合凋亡实验的分析和长时间的培养，故本研究的肺腺癌原代细胞用细胞株A549细胞替代。

## 1 材料与方法

### 1.1 临床资料

本研究自2008年3月-2008年9月之间，收集10例经临床和病理学诊断为肺腺癌的手术切除组织标本，其中男性6例，女性4例，年龄45岁-74岁，平均年龄61.7岁。病人从未接受过抗癌治疗。肺腺癌标本取材均经患者同意并获得武警浙江省总队医院医学伦理委员会的批准，根据WHO（2004）肺肿瘤组织学分类法和国际抗癌联盟（UICC）1997年修订的p-TNM分期标准包括肺腺癌Ⅱa+Ⅱb期10例（高分化4例、中分化6例）。10例肺腺癌病例按临床常规和WHO病理学标准进行肿瘤临床分期，见表 1。

**1:** 10例肺癌病人癌组织学特征 Characteristics of lung tumour tissues from 10 patients

Patient No.	Gender	Age (years)	Histological type (WHO)	pTNM
1	Male	74	Adenocarcinoma	pT3N2M0 G3
2	Female	70	Adenocarcinoma	pT2N2M0 G2
3	Male	69	Adenocarcinoma	pT3N2M0 G3
4	Female	45	Adenocarcinoma	pT2N2M0 G3
5	Female	53	Adenocarcinoma	pT3N3M0 G3
6	Male	62	Adenocarcinoma	pT3N3M0 G3
7	Male	72	Adenocarcinoma	pT3N3M0 G3
8	Male	58	Adenocarcinoma	pT3N0M0 G3
9	Male	54	Adenocarcinoma	pT3N2M0 G3
10	Female	60	Adenocarcinoma	pT3N2M0 G3

### 1.2 人肺腺癌细胞株A549及培养

无菌条件下，把人肺腺癌细胞株A549置于含有10%灭活小牛血清的RPMI-1640培养基（含100 U/mL青霉素和100 mg/L链霉素，pH值7.2-7.4）中，在37 ℃、5%CO_2_和相对湿度90%的培养箱中常规条件下孵育，隔日换液，取对数生长期的细胞用于试验。肺腺癌细胞株A549购自中国科学院上海生物化学及细胞生物学研究所细胞库。

### 1.3 药物及仪器

实验所用的DEX、CIS和四氮唑蓝为美国Sigma产品，二甲基亚砜（DMSO）由苏州正兴化工研究所分装提供，Annexin V FITC/PI细胞凋亡检测试剂盒购自上海贝博生物有限公司，细胞培养板、酶标板购自无锡耐思生物科技有限公司，台盼蓝购自上海碧云天生物技术有限公司。实验所涉及的仪器包括：SW-LZ-2FO超净工作台、Forma3111型CO_2_培养箱、Olympus CK40倒置相差显微镜、BIO-RAD-Model 550酶标仪、德国Partec PAS型流式细胞仪等。

### 1.4 原代肺腺癌细胞培养及DEX对CIS抑制肺腺癌细胞株A549生长的影响

取新鲜的肺腺癌手术切除的组织标本，撕碎后过100目铜网，滤过液300 g离心2 min，沉淀中加入20%灭活的小牛血清RPMI-1640培养液，37 ℃悬浮2 min-3 min，按上法离心，重复3次。然后用20%小牛血清RPMI-1640培养液重悬沉淀中的单个肺癌原代细胞。细胞悬液用0.5%台盼蓝染色，显微镜下检测活细胞数目为98%以上，再按上法离心后，用含20%灭活小牛血清的RPMI-1640配制细胞密度为5.0×10^5^个/mL的肺腺癌原代细胞悬液。96孔板每孔加100 μL原代细胞悬液，在37 ℃、5%CO_2_和相对湿度90%中培养。

在直径为3.0 cm细胞培养皿中，配制肺腺癌细胞株A549的细胞浓度为1×10^4^个/mL，于+/-DEX调至终浓度为1.0 μmol/L培养24 h，再经过+/-CIS 7 μmoL/L处理，并同空白对照组共4组培养2周和3周，培养条件为37 ℃、5%CO_2_和相对湿度90%。使用倒置显微镜拍照，并用0.5%台盼蓝染色，显微镜下记录活细胞数目，以对照组为100%，然后根据实验组活细胞数/对照组活细胞数×100%来计算不同实验组的细胞存活率。

### 1.5 肿瘤细胞生长增殖试验

在96孔细胞培养板中每孔分别接种细胞密度为5.0×10^5^个/mL肺腺癌原代细胞，分别加入DEX调至浓度为0.1 μmol/L、1.0 μmol/L、10.0 μmol/L和空白对照，在5%CO_2_、37 ℃和相对湿度90%条件中培养24 h。然后再以+/-CIS调至终浓度分别为7.0 μmol/L、17.0 μmol/L和34.0 μmol/L处理。各浓度均重复8孔。实验中以含20%灭活小牛血清RPMI-1640的等量肺腺癌原代细胞作为对照。再把上述培养板于5%CO_2_、37 ℃和相对湿度90%中培养48 h后，采用MTT比色法检测A550 nm吸收值，并计算出不同浓度的CIS下对经DEX处理过的肺原代细胞的生长率，计算公式为：生长率=试验组A550 nm值/对照组A550 nm值×100%。

### 1.6 流式细胞仪Annexin V-FITC/PI双标测定经CIS+/-DEX处理的肺腺癌细胞株A549的凋亡率

把细胞株A549按2×10^5^个/mL接种于6孔细胞培养板中，按空白对照组、CIS组（17.0 μmol/L）、DEX组（1.0 μmol/L）和CIS（17.0 μmol/L）+DEX（1.0 μmol/L）组培养48 h，每组设3个复孔。经细胞消化、收集，将细胞悬液以300 g离心3 min，弃上清液，用PBS离心清洗3次，然后细胞沉淀用100 μL结合缓冲液混悬，向细胞悬液加20 μL Annexin V-FITC混匀后避光1 h在冰上孵育，然后再加5 μL PI混匀后在冰上孵育5 min，再加500 μL结合缓冲液上Partec PAS型流式细胞仪检测，记录细胞凋亡值。

### 1.7 数据处理

采用SPSS 16.0统计学软件分析实验数据。肺癌细胞增殖试验A550 nm值以Mean±SD表示，组间比较采用*t*检验。

## 2 结果

### 2.1 DEX对CIS抑制肺腺癌原代细胞增殖效应的影响

10例肺腺癌原代细胞经CIS+/-DEX处理后，根据MTT测定的结果绘制细胞培养增殖曲线见图 1。结果显示，在10例中国人源的肺腺癌原代细胞中，CIS能明显抑制肺腺癌原代细胞增殖，但在联合DEX处理下，CIS抑制肺腺癌原代细胞增殖的效应受到了不同程度的抑制。

**1:**
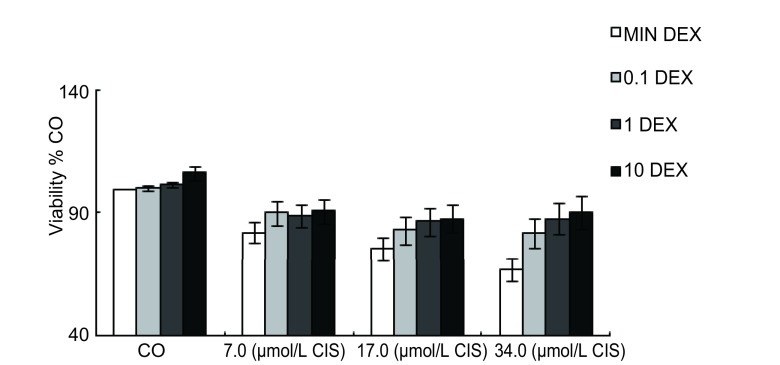
DEX对CIS抑制肺癌原代细胞增殖的影响（*n*=10） DEX promotes CIS-inhibited proliferation of primary lung carcinoma cells (*n*=10)

### 2.2 DEX降低CIS诱导的肺腺癌原代细胞死亡率和增加生存率

分离5号和10号病人被切除的肺腺癌的原代细胞并进行细胞培养，对培养2 d、4 d和6 d的细胞用0.5%台盼蓝染色后在显微镜下观测活/死细胞并计数，实验重复3次，结果如表 2显示。可以看出，DEX能减少CIS诱发肺腺癌原代细胞的死亡率，相对增加其生存率。

**2:** DEX促进细胞增殖或抑制细胞死亡（*n*=3） DEX promotes proliferation and inhibits CIS-induced death of fresh lung carcinoma samples (*n*=3)

Patients No.	Treatment	2d			4d			6d	
		Variable cells (×10^4^)	Dead cells (×10^4^)		Variable cells (×10^4^)	Dead cells (×10^4^)		Variable cells (×10^4^)	Dead cells (×10^4^)
No.5	CO	16.00±1.23	22.40±2.12		11.40±2.03	15.20±1.24		18.60±2.98	23.40±3.05
	DEX	16.00±2.03	23.60±1.79		14.00±1.76	13.40±2.23		21.20±3.22	21.80±3.21
	CIS	6.40±1.25	25.20±2.36		4.80±2.19	27.40±1.79		6.80±3.38	28.60±3.69
	CIS/DEX	12.40±2.51	25.00±1.87		5.40±1.53	17.80±2.45		11.60±1.16	24.80±2.36
No.10	CO	17.20±1.13	16.00±1.78		12.4±1.93	14.40±2.37		13.60±2.17	18.40±2.77
	DEX	17.00±3.11	16.20±2.23		13.6±2.02	12.20±2.87		14.60±1.78	17.8013.41
	CIS	7.80±2.11	22.40±3.11		6.60±1.98	23.20±2.47		7.20±2.96	20.60±3.12
	CIS/DEX	12.80±1.98	19.80±2.45		7.80±1.43	22.20±3.12		14.20±3.72	18.60±2.98

### 2.3 DEX干扰CIS抑制肺腺癌细胞株A549细胞增殖的影响

由于原代细胞不适合长时间的培养，本研究选用与肺腺癌相同的细胞株A549细胞替代原代细胞。图 2结果显示，单独接受CIS处理的A549细胞2周和3周后，出现大量细胞死亡；而CIS联合DEX处理的A549细胞，在2周和3周后细胞死亡率明显减少，相对细胞生存量较多，实验重复4次。表 3结果显示，单独接受CIS培养的A549细胞生存率仅在35%-37%之间，而CIS联合DEX处理下的A549的生存率却高达54%-67%，这说明DEX能减弱CIS诱导A549细胞的死亡效应。

**2:**
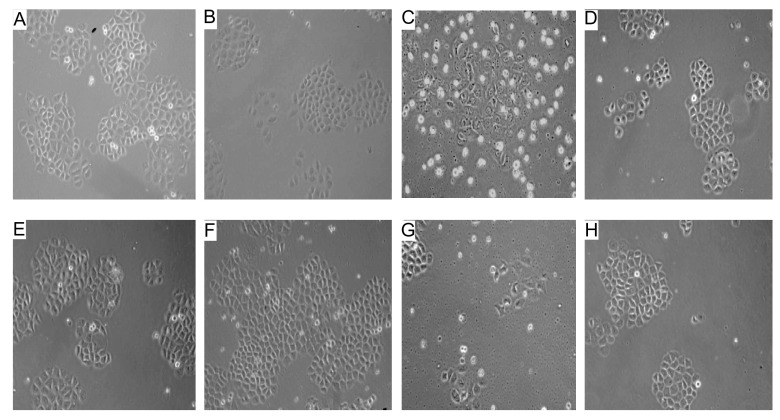
DEX对CIS诱导A549细胞增殖的抑制效应（×200） The inhibitory effect of DEX on CIS-induced A549 cell proliferation (×200)

**3:** DEX对CIS抑制A549细胞增殖率的影响 The effect of DEX on CIS mediated suppression of proliferation of A549 (*n*=4, Mean ±SD)

Drugs	Incubation time	
	2 weeks	3 weeks
-DEX, -CIS	100%	100%
1.0 μmol/L DEX	1 21.04%±5.52%^*^	131.32%±2.10%^*^
7.0 μmol/L CIS	35.22%±3.02%^*^	37.22%±2.39%^*^
1.0 μmol/L DEX+7.0 μmol/L CIS	67.17%±5.17%^*^	54.17%±1.94%^*^
Data are sampled from Fig 2. ^*^Comparing with -DEX, -CIS, *P* < 0.05.		

### 2.4 DEX对CIS抑制肺腺癌细胞株A549细胞凋亡效应的影响

在A549细胞培养48 h后，经流式细胞仪记录结果如图 3所示，QA1为早期凋亡细胞群，QA2为晚期凋亡细胞群，QA3为活细胞群，QA4是死亡细胞群。空白对照组、DEX组、CIS组和DEX+CIS组的早期凋亡率分别为4.9%、4.38%、44.09%和19.69%；晚期凋亡率分别为0.98%、0.6%、4.93%和3.68%。QA4是死亡细胞群，用以排除已死亡的细胞对受化疗药物引发凋亡的细胞检测结果的干扰，即排除假阳性。结果说明DEX能明显降低CIS诱导A549细胞的早、晚期凋亡效应。

**3:**
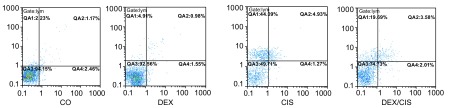
流式细胞仪测定CIS联合DEX处理肺腺癌细胞A549体外培养48 h的凋亡率 The apoptosis rate of A549 cells *in vitro* treated with CIS+/-DEX for 48 h by flow cytometry

## 3 讨论

铂类药物如CIS是临床上最为常用的抗癌化疗药物，CIS进入癌细胞后与细胞内DNA分子形成链内或链间交叉联接，导致DNA分子复制障碍，以此抑制肿瘤细胞的增殖^[9-11]^。糖皮质激素如DEX被引入肺癌辅助治疗，是由于它对淋巴细胞有着促凋亡和抗增殖的作用，还能减轻肺腺癌化疗引起的毒、副反应和肺腺癌自身引起的其它不良症状。中国人肺腺癌接受化疗时，一般采用DEX协同铂类抗癌药物如CIS治疗^[12]^。国内有研究^[13]^小组利用小鼠动物模型就缺氧诱导因子-1α对肺细胞生长、分化及对血管内皮生成因子等的表达进行了系列研究，并通过DEX降低胞外信号调控激酶介导的信号转导来抑制缺氧诱导因子-1α的表达，其抑瘤机制是DEX通过诱导MKP-1增加从而降低了VGEF的表达，减弱了有助肿瘤滋养的血管生长，因此产生抑制癌组织生长的可能。国内有综述^[14]^报道糖皮质激素有助于肺癌的化疗效应，其理由是糖皮质激素增加了细胞内P21和P27的表达，以此提高了P53对细胞周期的调控，引发肿瘤细胞的凋亡。

但有研究^[6]^表明肺腺癌细胞株P693细胞在CIS联合DEX处理后，DEX不仅能降低细胞的凋亡蛋白CD95L、caspase-9等的基因表达，而且还增加了抗凋亡蛋白BCL2和*XIAP*基因的表达，因此，DEX抑制了化疗药物CIS诱导的肿瘤细胞的凋亡。令人感兴趣的是，糖皮质激素如何通过介导肿瘤细胞对肿瘤化疗产生抑制作用。有研究^[15]^证实这与肿瘤细胞内糖皮质激素受体的特异性相关，其一种解释是在不同类型细胞内受体存在共同激活或抑制作用的差异性。Wu等^[16]^在比较乳腺癌细胞株的基因表达中发现，DEX通过增加MKP-1蛋白的表达，降低CIS诱导细胞内JNK蛋白活性表达而消弱细胞凋亡的效应，其机制涉及不同基因序列调控位点、细胞内信号的转导、代谢、细胞周期和DNA的修复等方面，最终能使凋亡蛋白的最终信号cleaved caspase-3片段表达明显下降。另外，对于肺腺癌治疗，糖皮质激素能否诱导细胞凋亡或增殖，肺癌患者存在病因、种族和组织等的差异性，可能与细胞特异性的转录调控有关^[8]^。

本研究中，10例经病理学确诊为肺腺癌患者的原代细胞，接受不同浓度的CIS联合DEX处理，通过MTT方法检测肺腺癌原代细胞的增殖状况，图 1结果显示DEX可降低细胞增殖受CIS抑制的效应。在肺癌组织的5号和10号标本中分离出的肺腺癌原代细胞，单独接受CIS处理的实验组出现大量的细胞死亡；与CIS联合DEX处理的实验组相比，其结果如表 2所示，DEX能减少CIS引发的死亡量。由于肺腺癌原代细胞不适合长时间的培养和细胞凋亡率的检测，本实验选用了人类肺腺癌的A549细胞株代替原代细胞进行了研究，结果如图 2和表 3所示，单独接受CIS处理的A549细胞2周后，细胞死亡率达65%左右，3周后细胞死亡率则降低；相比CIS联合DEX处理的A549细胞，在2周和3周后细胞死亡率明显降低。同样，用流式细胞术检测结果如图 3显示DEX能明显降低CIS诱导的细胞凋亡率。

近期有研究^[17]^表明糖皮质激素能诱发增加肺腺癌细胞株A549的人类多耐药基因*MRP3*的高表达，使得肺腺癌细胞产生化疗耐药。因此，结合本实验的研究结果，提示有必要在中国人种的肺腺癌治疗中再进行大量的类似本实验研究，确定糖皮质激素对中国人群的肺腺癌化疗影响是否存在着广泛地降低CIS抗癌效应，或者采用非类固醇类的药物协同抗癌药物进行化疗，以比较联合糖皮质激素的化疗效应。
